# The Future of HCV Therapy: NS4B as an Antiviral Target

**DOI:** 10.3390/v2112481

**Published:** 2010-11-10

**Authors:** Hadas Dvory-Sobol, Philip S. Pang, Jeffrey S. Glenn

**Affiliations:** 1 Department of Medicine, Division of Gastroenterology and Hepatology, Stanford University School of Medicine, CCSR 3115A, 269 Campus Drive, Palo Alto, CA, USA; E-Mails: hadasds@stanford.edu (H.D.-S.); pspang@stanford.edu (P.S.P.); 2 Division of Infectious Diseases and Geographic Medicine, Stanford University School of Medicine, 300 Pasteur Drive, Stanford, CA, USA; 3 Veterans Administration Medical Center, 3801 Miranda Avenue, Palo Alto, CA, USA

**Keywords:** HCV, hepatitis C virus, antiviral agents, NS4B, clinical trials, RNA binding, amphipathic helix, NTPase

## Abstract

Chronic hepatitis C virus (HCV) infection is a major worldwide cause of liver disease, including cirrhosis and hepatocellular carcinoma. It is estimated that more than 170 million individuals are infected with HCV, with three to four million new cases each year. The current standard of care, combination treatment with interferon and ribavirin, eradicates the virus in only about 50% of chronically infected patients. Notably, neither of these drugs directly target HCV. Many new antiviral therapies that specifically target hepatitis C (e.g. NS3 protease or NS5B polymerase inhibitors) are therefore in development, with a significant number having advanced into clinical trials. The nonstructural 4B (NS4B) protein, is among the least characterized of the HCV structural and nonstructural proteins and has been subjected to few pharmacological studies. NS4B is an integral membrane protein with at least four predicted transmembrane (TM) domains. A variety of functions have been postulated for NS4B, such as the ability to induce the membranous web replication platform, RNA binding and NTPase activity. This review summarizes potential targets within the nonstructural protein NS4B, with a focus on novel classes of NS4B inhibitors.

## 1. Introduction

Hepatitis C virus (HCV) infects an estimated 170 million people around the world [1]. Infection often leads to cirrhosis and sometimes to hepatocellular carcinoma [2,3]. No vaccine is currently available. The only FDA approved regimen relies on a combination of subcutaneously administered pegylated interferon-α (IFN) and oral ribavirin (RBV). These non-HCV specific antivirals, however, have limited efficacy. A sustained virologic response (SVR), defined as the absence of HCV RNA in the serum six months after the cessation of HCV therapy, can be achieved in only about 42% to 53% of patients with HCV genotypes 1 and 4, respectively, and up to 78% to 82% of patients infected with HCV genotypes 2 or 3 [4,5]. These genotype specific differences are hypothesized to be due in part to the evolution of anti-immune factors [6], which limit the immune response generated by exogenous interferon. Furthermore, because the majority of patients infected with HCV in the United States are infected with genotype 1, the most treatment-refractory genotype, nearly half of all patients treated for HCV with IFN/RBV therapy will fail treatment. IFN/RBV combination therapy is also expensive, and results in serious side effects such as fever, fatigue, anemia, leucopenia, thrombocytopenia, and depression [7–9].

For these many reasons, there is an urgent need for HCV-specific antivirals that are both more efficacious and tolerable. Furthermore, given the ability of HCV to rapidly acquire resistance to antivirals, as has been already shown in multiple clinical trials [10–13], anti-HCV therapeutics that target multiple aspects of the HCV-life cycle are expected to be needed in order to control this infection—as is currently the case for HIV. The rapidity with which HCV acquires resistance is due in part to the fact that the HCV NS5B polymerase lacks a proofreading function, which results in an estimated erroneous base substitution rate of ∼1 in 10,000 replicated bases [14]. Practically, this means that every copy of HCV that is made contains at least one mutation. Given the massive number of HCV virions produced in patients chronically infected with HCV (1.3 × 10^12^ virions per day) [15], every patient is therefore best considered as infected with a population of HCV quasi-species [16,17]. Such quasi-species, when under selective antiviral pressure, lead to the emergence of resistant strains.

Therefore, a collection of well-tolerated HCV antivirals that target multiple steps of the HCV life cycle, and which thereby can overwhelm the ability of such quasi-species to adapt, represents the idealized goal for future HCV drug development. These agents are termed “specifically targeted antiviral therapy for HCV” or STAT-C drugs [18,19]. Among the most promising new anti-HCV agents in clinical development are those that target the NS3 protease, the NS5A protein, the RNA-polymerase NS5B, and compounds that directly inhibit HCV replication through interaction with host cell proteins (see [20–23] for more in depth reviews of these agents). Another new promising strategy consists of targeting the NS4B protein. This review focuses on the recent developments of potential antivirals against targets within NS4B.

## 2. Important features of NS4B

**Figure 1.**
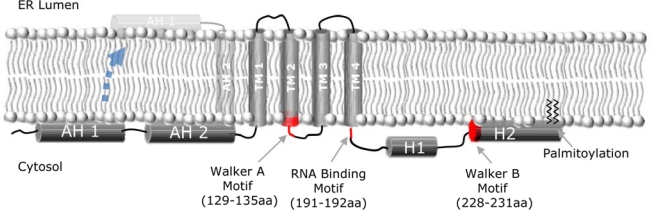
NS4B membrane topology. Schematic representation of the HCV NS4B protein, and its proposed topology with respect to the ER membrane, depicting the N-terminal amphipathic helices (AH1, AH2), the four transmembrane domains (TM1-4), and the two C-terminal helices (H1, H2). A fraction of NS4B appears to be able to undergo a post-translational translocation event wherein the N-terminus of NS4B adopts a luminal orientation (indicated by dashed blue arrow), thereby creating a fifth transmembrane domain. Walker A and B motifs and RNA binding motif are also indicated.

NS4B is a relatively poorly characterized 27 kDa protein with at least four predicted transmembrane (TM) domains [24–26] (Figure 1). As a consequence of polyprotein processing by the NS3-4A protease, the N- and C-terminal parts of NS4B are believed to be oriented towards the cytosolic side of the endoplasmic reticulum (ER) membrane. An amphipathic helix (AH), a helix in which one side is hydrophobic and the other hydrophilic, is located within the first 27 amino acids of NS4B and designated AH1 [27]. When N-terminally fused to a construct containing GFP, AH1 was shown to mediate membrane association, and point mutations disrupting its hydrophobic face abrogated membrane association [27]. Of note, fusion of amino acids 1 to 29 or 1 to 40 of NS4B did not confer membrane association [28], possibly reflecting differences in the respective expression constructs. Insertion of artificial glycosylation acceptor sites at various positions in NS4B yielded evidence in support of the predicted ER luminal loops around amino acid positions 112 and 161 [24,29]. In addition, a fraction of NS4B may acquire a fifth TM domain, postulated to result from post-translational translocation of the N-terminal domain into the ER lumen (see Figure 1) [29].

Recently, we and others have identified a second AH (AH2) immediately following AH1 (Figure 1) that is sufficient to confer tight membrane association and is required for viral replication [28,30,31]. It is suggested that this amphipathic α-helix has the potential to traverse the phospholipid bilayer as a transmembrane domain [28]. We have shown that AH2 can both oligomerize with itself and specifically mediate lipid vesicle aggregation [30]—a function that might be relevant for the role of NS4B in forming the membranous web replication platform [32] (discussed below). A variety of biophysical measurements have confirmed the predicted helical nature of AH1 [33] and AH2 [28,30].

Secondary structure analyses have also predicted two helices in the C-terminal portion of NS4B. The first of these C-terminal helices (H1) is composed of amino acids ∼200–213 and is highly conserved among HCV genotypes. The second helix (H2), also confirmed by CD [31], is composed of amino acids ∼229–253 and is more variable. H2 also has an amphipathic structure, mediates membrane association, and is involved in the formation of functional HCV replication complexes [26,28,31,32,34–38]. NS4B has also been shown to interact with itself, suggesting an ability to form homo-multimers [39,40]. Finally, NS4B has been shown to be palmitoylated at two C-terminal cysteine residues [40]. It is hypothesized that this palmitoylation plays an important role in NS4B oligomerization. However, the role of C-terminal palmitoylation of NS4B in the HCV life cycle remains to be further explored.

One of the more striking NS4B functions identified to date is its reported effect on intracellular membranes. Expression of the HCV NS4B protein alone is sufficient to cause formation of the so-called “membranous web” structure [32,37] which is thought to represent the platform upon which HCV replication takes places. Like all positive strand RNA viruses, HCV replicates its genome in intimate association with intracellular membranes. The membranous web is believed to be derived in part from the ER. A number of studies have also suggested that the early endosome proteins Rab5 and Rab7 [41,42], phosphatidylinositol 4-kinase III alpha [43] and autophagy protein ATG5 [44] may play a role in HCV genome replication or membranous web formation. The membranous web consists of a collection of vesicular-like structures detectable by electron microscopy. Under light microscopy, the membranous web is believed to be correlated with so-called membrane associated foci (MAF) [45]. Both N- and C-terminal amphipathic helices have been implicated in the formation of such foci [28,31]. The mechanism(s) by which NS4B induces the membranous web, and which host cell components are hijacked for this purpose are still largely unknown.

Site-directed mutagenesis in the replicon and HCVcc systems has demonstrated the essential role of NS4B in HCV RNA replication [24,27,28,31,38,46,47]. Jones *et al.* [38] revealed an additional role of NS4B in viral assembly when a single amino acid substitution, N216A, in NS4B was shown to be sufficient to increase the titer of JFH1 virus by five-fold without affecting HCV RNA replication. This highly conserved position, located between helices H1 and H2, might be critical for interaction between NS4B and other components of the viral assembly machinery.

Physical interactions between NS4B and other nonstructural proteins have been demonstrated by glutathione S-transferase pull-down experiments [39]. In addition, intragenotypic interactions between NS3 and NS4B were demonstrated in the replicon system [48], which may underlie recent findings of synergy between pharmacologic inhibitors of NS3 and NS4B (see Section 3.2. below).

Another functionality of NS4B was identified by Einav and colleagues, who discovered a functional nucleotide-binding motif (NBM) within NS4B. This motif was found to mediate GTP hydrolysis and is essential for HCV replication [49]. Thompson *et al*. confirmed this finding recently and also demonstrated ATPase and adenylate kinase activity for NS4B [50].

Finally, NS4B has been shown to bind the viral RNA, an interaction that is critical for HCV replication [51]. These aspects of NS4B functionality are discussed further, below.

## 3. NS4B as an antiviral target

NS4B is particularly difficult to study due to its integral membrane association. In the following sections, we will summarize the potential of some of the above-described features of NS4B as antiviral targets and describe recent advancements in the development of new pharmacological approaches for inhibiting NS4B functions.

These pharmacologic inhibitors derive in large part from the above identified functions of NS4B. As our knowledge of the latter increases, this list is expected to grow.

### 3.1. NTPase activity

NS4B has been shown to contain a nucleotide binding motif (NBM), with the Walker A motif (129GSIGLK135) located between transmembrane domains 2 and 3, and the Walker B motif (228DAAA231) located in a central portion of the C-terminus [49,50]. This NBM binds and hydrolyses GTP and ATP. The NS4B NBM has also been shown to mediate an adenylate kinase activity, which catalyzes the synthesis of ATP and AMP from two ADP molecules [50]. The precise role of NS4B’s NTPase activity in the HCV life cycle, however, remains unknown, although it has been suggested that NS4B’s GTPase activity might play a role in NS4B-induced cellular transformation and tumor formation [52]. The NS4B NBM motif is conserved across all HCV genotypes and isolates, highlighting its importance for HCV infectivity *in vivo*. Introduction of point mutations with the NBM can dramatically inhibit RNA genome replication [49], providing strong genetic validation that the NBM represents an attractive potential target for anti-HCV therapy. One so-called adaptive mutation in the NS4B NBM (K135T) has been reported to enhance replication [53]. While the effect of this mutation on NS4B GTPase activity has not been determined, it is interesting to note that many host GTPases have a highly conserved T at the corresponding adjacent position of their Walker A motif (GX_1_X_2_X_3_X_4_GKT), whereas the unadapted wild-type NS4B does not (e.g. amino acid 136 is a V in the genotype 1b replicon) [49].

The specificity of NS4B for ATP *versus* GTP is another interesting area of investigation. Einav *et al*. found NS4B to have a preference for GTP binding [49], while Thompson *et al*. found ATP to be its more active hydrolysis substrate [50]. Thompson *et al*. showed slow hydrolysis of GTP and suggested that this represents the intrinsic hydrolysis of the enzyme and that a yet unknown NS4B GTPase activating protein (GAP) [50] might stimulate catalysis.

The HCV NBM has features that distinguish it from host cell GTP-binding and hydrolyzing proteins. In particular, while the amino acids within the NS4B NBM are similar to those found in the NBMs of host GTPases, the amino acids immediately flanking the NS4B NBM are highly conserved across HCV isolates, yet very different from those found in host cell GTPases [49]. Moreover, T221 of NS4B, which corresponds to the so-called PM2 motif that has been implicated in chelating a Mg++ ion in available structures of host GTPases, can be mutated without apparent effect on HCV replication [54], suggesting that the co-factor requirements for NS4B may be different than for host cell GTPases. Thus, pharmacologic inhibition of the enzymatic activity encoded in the NS4B NBM might be achievable with a sufficiently favorable therapeutic index. Such an inhibitor might also have the additional benefit of potentially reducing malignant transformation, and could be relevant to the hepatocellular carcinoma associated with HCV.

### 3.2. RNA binding

NS4B has been found to bind the 3’ terminus of the negative strand of the HCV genome [51]. One means by which this was demonstrated involved the use of a microfluidics device in combination with a mechanical button that enhances the detection of intermolecular interactions [51]. This button system is known as MITOMI: the mechanical trapping of molecular interactions [55]. Advantages of this method include the requirement for very small sample volumes and the ability to express the protein in the presence of microsomal membranes, which enables membrane proteins to adopt a more natural folding.

Using this method, a Kd for NS4B RNA binding was determined to be ∼3 nM, with a preference for the 3’ terminus of the negative sense HCV RNA. This region is predicted to harbor a highly conserved secondary structure that is presumably recognized by NS4B. Moreover, specific binding to this region suggests that NS4B may play a role in the initiation of progeny plus strand RNA genomes that begin their transcription at the 3’ terminus of the negative strand. Highly conserved arginine residues within NS4B were predicted to mediate NS4B’s RNA binding activity, and this hypothesis was tested by determining the effect of mutating specific arginines to alanines. Such mutations could both abrogate RNA binding and HCV genome replication, highlighting the importance of this activity for the viral life cycle and providing genetic validation of the potential of NS4B’s RNA binding activity as an antiviral target.

Einav and colleagues then used the above microfluidics platform to perform a high-throughput screen for small molecules capable of inhibiting the binding of NS4B to HCV RNA. This screen identified the small molecule clemizole hydrochloride, which inhibited NS4B-HCV RNA binding with an IC_50_ of ∼24 nM. Cellular-based replication assays subsequently demonstrated the ability of clemizole to inhibit HCV replication [51]. Clemizole’s antiviral effect is notably modest, with an EC_50_ of 8 μM against HCV genotype 2a. Interestingly, when combined with some of the NS3 protease inhibitors in most advanced clinical development (*i.e.*, telaprevir and boceprevir), clemizole exhibits dramatic *in vitro* synergy [56]. This is in contrast to most combinations of anti-HCV agents, which generally exhibit additive interactions. The synergy between clemizole and a protease inhibitor was found to decrease the emergence of resistant mutations without conferring cross-resistance [56] in the HCV replicon system. It is speculated that an interaction between NS4B and NS3, perhaps involving a conformational change, is one reason for the observed dramatic synergy between clemizole and the NS3 protease inhibitors.

If the above *in vitro* synergistic effects are operative *in vivo*, such clemizole-protease inhibitor combinations may represent an ideal foundation for future interferon-free anti-HCV cocktails. Another attractive implication of such synergy is the potential to use these protease inhibitors at lower doses in patients, where the desired antiviral and anti-resistance effects could be maintained, while decreasing the incidence of important toxicities associated with the protease inhibitors (such as severe rash and anemia).

Clemizole hydrochloride is a H1 histamine receptor antagonist that was widely used in the 1950–60s to treat allergic disorders. One of its hallmarks is that it is very well tolerated [57]. Because of its prior excellent safety record, the re-purposing of this drug for treatment of chronic HCV is being actively pursued in clinical trials (e.g. NCT00945880).

Finally, the search for clemizole derivatives with increased potency is an active area of research, with a number of analogs with increased antiviral activity having already been identified [58].

### 3.3. Lipid vesicle aggregation and 4BAH2 oligomerization

Because formation of the membranous web is essential for HCV replication, but dispensable for the host cells, disrupting the molecular machinery employed to establish the membranous web represents an attractive potential therapeutic target. As stated above, recent work suggests that a second amphipathic helix (AH2) exists within the N-terminus of NS4B [28,30]. This AH has been designated (4BAH2) [30]. It spans amino acids 43 to 65 [30] or 42–66 [28] and plays an important role in this process. 4BAH2 is notably conserved across all HCV genotypes and isolates. Studies of the interaction of 4BAH2 with lipid vesicles composed of 1-palmitoyl-2-oleoyl-sn-glycero-3-phosphocholine (POPC) demonstrate that 4BAH2 mediates both helix oligomerization and specific aggregation of lipid vesicles (for example, no such activity was observed with a peptide corresponding to the NS4B AH1 amphipathic helix). The structures formed by this vesicle aggregating activity are reminiscent of the membranous web [30], and we hypothesize that the activity encoded in 4BAH2 may represent a key component of the molecular machinery employed by NS4B to establish the membranous web. This ability of 4BAH2 to cause lipid vesicle aggregation was also exploited to identify small molecule inhibitors of 4BAH2-mediated vesicle aggregation, using a novel high-throughput screen. In this screen, a synthetic peptide comprising the 4BAH2 sequence was added to fluorescently labeled lipid vesicles in 384-well plates, wherein each well contained a different member of a small molecule library. The vesicle aggregating activity of 4BAH2 was monitored by automated fluorescent microscopy. Pattern recognition software was then used to quantitate the amount of fluorescent signal contained within 4BAH2 induced lipid vesicle aggregates. In this manner, several candidate pharmacologic inhibitors of lipid vesicle aggregation were indentified. The specificity of their inhibitory activity was confirmed in secondary assays that were based on monitoring aggregate size using dynamic light scattering (DLS), in the presence or absence of individual compounds. The most potent inhibitory molecules in the DLS assay were then subsequently found to inhibit HCV genome replication with EC50s in the nanomolar range [30]. Two compounds were further studied in detail using quartz-crystal microbalance and dissipation to monitor the effects of compounds on the membrane association of 4BAH2, and atomic force microscopy to monitor the compounds’ effects on 4BAH2 oligomerization. Interestingly, at least two different mechanisms of inhibition could be defined: one compound (“C4”--5-(N-Methyl-N-isobutyl)amiloride) was able to inhibit 4BAH2 oligomerization while another (“A2”--7-[chloro(difluoro)methyl]-5-furan-2-yl-N-(thiophen-2-ylmethyl)pyrazolo[1,5-a]pyrimidine-2-carboxamide) was found to inhibit the ability of 4BAH2 to associate with membranes [30]. Importantly, clemizole was found to have no activity in the 4BAH2 vesicle aggregation assay, and the 4BAH2 inhibitors were found to have no effect on NS4B RNA binding—suggesting that 4BAH2 inhibitors and NS4B RNA binding inhibitors represent two distinct classes of NS4B inhibitors, with different mechanisms of action. Thus, each class can be independent components of future anti-HCV cocktails. Consistent with this is the observation that, *in vitro*, clemizole is synergistic with 4BAH2 lipid aggregation inhibitors [58]. Gouttenoire *et al.* [28] have also suggested that the 4BAH2 segment is likely associated with oligomerization. Moreover, their study suggested that this helix is capable of traversing the phospholipid bilayer as a transmembrane domain. Because all of 4BAH2 is initially cytosolically oriented, C4 and A2 may thus inhibit the majority of 4BAH2 that remains on the cytosolic side of the ER membrane in the presence of other NS proteins [29] as well as the fraction of 4BAH2 that might be destined for more profound interactions with the membrane. Finally, we postulate that just as therapeutics that target AH2’s functionality inhibit HCV replication, therapeutics that target AH1 and disrupt its membrane interaction activity will inhibit HCV replication.

## 4. Conclusions

The functions of NS4B identified to date have enabled the development of two new classes of anti-HCV drugs: one class that interferes with the ability of NS4B to bind HCV RNA; and another that inhibits NS4B’s interactions with membranes. Notably, preliminary data suggests that the drugs that target these individual functions of NS4B are synergistic. It is expected that these inhibitors will be used as a basis for future structure-function studies designed to yield more potent derivatives and eventual new clinical candidates. Furthermore, as other functions for NS4B have already been identified, (e.g. its ability to hydrolyze NTPs), it is hoped that these too can be targeted by separate classes of antivirals.

Knowledge of the molecular virology of HCV, and the functions of its encoded proteins, has allowed for the design of new drugs that directly target HCV. Other than safety and efficacy, the major challenges of HCV drug development are the ability to design therapeutics that inhibit all HCV genotypes and yet are insensitive to the emergence of drug mutants. One solution to this latter challenge is to administer a cocktail of antiviral agents that target different functions in the viral cycle. Accordingly, most current clinical trials test a combination of antiviral agents that combine STAT-C agents with IFN or RBV. As more and more STAT-C agents emerge, and more efficacy data is accumulated, future studies that are able to omit or decrease IFN or RBV may be considered—such an approach would hopefully provide even more tolerable and efficacious therapies to fight HCV and its complications.
